# Extended anticoagulation for VTE: what evidence justifies it?

**DOI:** 10.3389/fphar.2023.1241979

**Published:** 2023-08-29

**Authors:** Anushka Walia, Vinay Prasad

**Affiliations:** ^1^ School of Medicine, University of California, San Francisco, San Francisco, CA, United States; ^2^ Department of Epidemiology and Biostatistics, University of California, San Francisco, San Francisco, CA, United States

**Keywords:** anticoagulation, venous thromboembolism, pulmonary embolism, deep venous thromboembolism, clot

## Opinion

Venous thromboembolism (VTE), which includes pulmonary embolism and deep vein thrombosis, is associated with significant morbidity and mortality. The cornerstone of VTE treatment is anticoagulation with direct oral anticoagulants (DOACs), vitamin K antagonists, or heparin. Acute VTE is treated with 5–10 days of active therapy followed by extended anticoagulation of various durations. For VTE with known transient provoking factors, anticoagulation is discontinued after 3–6 months (Ortel et al., 2020). In the case of unprovoked VTE, anticoagulation is typically continued indefinitely. These guidelines are based on clinical studies demonstrating a persistent risk of VTE recurrence in patients with first unprovoked VTE, as well as loss of prophylactic effect once treatment is stopped (Couturaud et al., 2015). However, direct advantage of indefinite over short-term anticoagulation has not been assessed in randomized clinical trials (RCTs), and must be carefully weighed against the risk of major bleeding events. In this commentary, we explore the challenges of assessing the true value of indefinite anticoagulation in patients with first time VTE without identifiable provoking risk factor.

### Provoked vs. unprovoked VTE

Distinguishing between provoked and unprovoked VTEs is imperfect and often challenging. 2016 recommendations by the International Society on Thrombosis and Haemostasis (ISTH) define transient provoking factors for VTE as those associated with a “greater than 3-fold increased risk of first VTE or half the risk of recurrent VTE after stopping anticoagulant therapy” (Kearon et al., 2016). Provoking factors range from known major triggers such as surgery and extended hospitalization to minor triggers like acute medical illness or estrogen therapy. Because identification of risk factors is often contingent on history and clinician judgement, the distinction between provoked and unprovoked VTEs is not black or white and definitions differ by study (Iorio et al., 2010). Yet, it determines whether a patient will receive therapy for a few months or the remainder of their life.

There is limited data comparing long-term VTE recurrence rates among patients with provoked and unprovoked VTEs, and results have been mixed. The prospective GARFIELD-VTD study, which included 10,207 patients with VTE worldwide, compared outcomes in the presence or absence of transient provoking factors (Ageno et al., 2021). No difference in rates of recurrent VTE or mortality was found between provoked and unprovoked groups (4.4 vs. 2.9 per 100 person-years and 3.7 vs. 3.4 per 100 person-years respectively). This may be partially due to the longer duration of anticoagulation in the unprovoked group, with 51.5% of patients with unprovoked VTE and 36.7% of patients with transient provoking factors remaining on anticoagulation at 12 months. Iorio et al. found that the 24-month VTE recurrence rate after stopping anticoagulation was 2.3-fold higher in patients with unprovoked first VTE than patients with VTE provoked by a transient risk factor (Iorio et al., 2010). Recurrence rates varied depending on whether transient provoking factors were surgical or non-surgical. A 2003 study on recurrent VTE by Baglin et al. found similar results (Baglin et al., 2003). Still, it is not clear whether long term outcome differences between risk groups are high enough to warrant such divergent treatment approaches.

Clinically unsuspected or “incidental” VTEs represent another major clinical challenge, as their clinical significance has never been proven. The threshold to detect clots is lower today than in the past, with VTEs commonly found on routine CT scans. Rates of clinically unsuspected VTEs are as high as 3.3% in patients referred for chest CT according to one meta-analysis, and detection largely depends on the skill of the radiologist (Chiu and O’Connell, 2017). Despite the increased rates of diagnosis of VTEs over the years, the mortality rate has not increased. Current guidelines recommend the same treatment for clinically suspected and unsuspected VTEs, based on retrospective data suggesting an association between incidental VTEs and increased risk of future events. A recent multicenter prospective trial by examined 90-day VTE recurrence rates in patients with isolated subsegmental pulmonary embolism (SSPE) managed without anticoagulation (Le Gal et al., 2022). They found that despite 8 recurrence events among 266 patients, no patients had a fatal recurrent pulmonary embolism. The clinical benefit of anticoagulation in patients with SSPE is unclear, and the same may be true for other incidental VTEs.

### Recurrent VTE vs. major bleeding

Anticoagulation trials typically use a primary endpoint of VTE recurrence and a safety endpoint of bleeding. The clinical decision of how long to anticoagulate rests on a precarious balance between these possibilities, which may not be equivalent risks. While recurrent VTE may carry significant mortality risk, the case fatality rate (CFR) for major bleeding has been demonstrated to be higher than the CFR for VTE recurrence among patients receiving anticoagulants. One metanalysis of 68 clinical studies, of which 56 were RCTs, found CFRs of major bleeding and recurrent VTE to be comparable during 6 months of anticoagulation (Carrier et al., 2010). However after the initial 3–6 months of treatment, the CFR of recurrent VTE was about 3 times lower than that of major bleeding. In a study on the RIETE database, a prospective registry for patients treated for VTE, CFRs of recurrent VTE and major bleeding were 12.1% and 19.7% respectively during anticoagulant therapy (Lecumberri et al., 2013). While the CFR of recurrent VTE decreased from 16.1% during the first 3 months of treatment to 2.0% after 3 months, the CFR of major bleeding only changed from 20.2% to 18.2%.

Because bleeding risk typically increases with age while rates of VTE recurrence peak immediately after the first event and drop to a plateau after a few years, the benefit/risk ratio of anticoagulation may decline over time (Bounameaux and Perrier, 2008). Importantly, bleeding risk may be underestimated in clinical trial populations, as real-world patients are older and have greater comorbidities, including higher rates of reduced renal function—a risk factor for major bleeding (Geldhof et al., 2014).

While physicians may halt anticoagulation in patients at high risk of major bleeding, this risk is difficult to assess. None of the available bleeding risk assessment tools have been validated in clinical trials, and several studies have demonstrated that they have poor predictive value for patients with VTE (Vedovati et al., 2020). Partly due to the inability to predict major bleeding, it is unclear whether to prioritize the risk of VTE recurrence or the risk of hemorrhage. Although clinicians have traditionally leant towards the former, long-term data comparing mortality associated with VTE vs. hemorrhage is necessary to make this decision. The risk-benefit profile of anticoagulants must also be re-evaluated in the era of DOACs, which have become first-line for most patients with VTE over vitamin K antagonists.

### Mortality and RCTs

Whether anticoagulation yields improvements in patient-centered outcomes such as survival and quality of life is unclear. Although CFRs can provide some information on mortality, answering this question conclusively requires adequately powered RCTs with long follow-up periods. A Cochrane metanalysis of 11 clinical studies comparing short-term and prolonged treatment with vitamin K antagonists found that prolonged therapy was associated with a strong reduction in recurrent VTEs (Middeldorp et al., 2014). However, notably, no significant reduction in mortality in those who received shorter treatment was found (RR 0.89, 95% Cl 0.66 – 1.21, *p* = 0.46) but prolonged treatment was associated with a substantially increased bleeding complications (RR 2.60, 95% Cl 1.51 – 4.49). In addition, their analysis did not demonstrate “rebound” hypercoagulability post-therapy (Cundiff, 2008) that leads to fear of discontinuing treatment.

RCTs of anticoagulants have not provided information on the optimal duration of therapy for VTE prophylaxis after first-time unprovoked VTE. One trial compared outcomes 6 months of oral anticoagulant therapy with indefinite anticoagulation in 227 patients with a second episode of VTE (Schulman et al., 1997). During 4 years of follow-up, authors found a significantly increased risk of recurrent VTE in the 6-month group but no difference in mortality between the two groups. In fact, no cases of fatal pulmonary embolism were confirmed. The relative risk of major hemorrhage in the 6-month group compared to the indefinite anticoagulation group was 0.3, and 3 hemorrhages in total were fatal.

No trial has directly compared 3–6 months of anticoagulation with indefinite treatment after first-time unprovoked VTE, and the longest period of treatment in the extended arm of an RCT has been around 4 years. Despite lack of evidence, patients may remain on anticoagulant therapy for decades.

### Conclusion

The secondary anticoagulation strategy for unprovoked VTE presents several challenges. Differentiating unprovoked and provoked VTE is often subjective and many cases fall in a gray area between these categories. Prolonged anticoagulation carries a persistent high risk of major bleeding that may be poorly estimated and comparable with the risk of recurrent VTE. Data comparing lifetime risks of bleeding and recurrent VTE is lacking. In addition, RCTs have not established a survival or quality of life benefit of indefinite anticoagulation or directly compared indefinite and short-term therapy. With an aging population and increased PE detection capability, rates of anticoagulation are expected to increase, adding costs to our healthcare system.

The evidence base for indefinite thromboprophylaxis must be increased. Until we have more data, a patient-centered approach that evaluates individual risk factors and preferences should guide anticoagulation after VTE.
